# Coronavirus disease 2019 pandemic: Do not forget patients with severe mental illness

**DOI:** 10.1177/0020764020939982

**Published:** 2020-07-07

**Authors:** S. Bentolhoda Mousavi

**Affiliations:** Psychosis Research Center, University of Social Welfare and Rehabilitation Sciences, Tehran, Iran

Pandemics, such as coronavirus disease 2019 (COVID-19), cause substantial fear, insecurity, and uncertainty (Asmundson & Taylor, 2020). This could be more prominent in people with severe mental disorders such as schizophrenia or bipolar disorder as having a prior history of psychiatric disorders can be a risk factor for increased psychological distress after going through any disaster-related traumatic experience (Alvarez & Hunt, 2005; Cukor et al., 2011). It is not surprising that people with history of mental disorders would need further support during pandemics (Brooks et al., 2020). Working in the field, I have observed that patients with severe mental illnesses and their care givers express several concerns about the current pandemic of COVID-19 that should be addressed carefully. Here I have classified these concerns into three main groups: (a) Whether this infection induce any severe psychiatric disorder or can it trigger preexisting conditions? (b) What are the main obstacles of treatment during this pandemic? (c) And what can be done?

The first mentioned concern is whether the virus is associated with increased incidence of psychiatric disorders. In this case, a bio-psycho-social explanation can be insightful. There is anecdotal evidence to link the family of corona virus such as influenza with the incidence or exacerbation of bipolar disorder, suicide, and psychosis (Okusaga et al., 2011; Severance et al., 2011). One study, for instance, showed association of seropositivity for influenza A, B and coronaviruses with the history of mood disorders and seropositivity of influenza B with previous suicide attempt and psychosis (Okusaga et al., 2011). Evidence of neuro invasion has been also found in previous studies. Both the related immune reactions and the direct impact on brain can explain the potential association with mood disorders, psychosis and suicide (Arbour et al., 2000; Dessau et al., 2001; Thomas & Haider, 2020). The data, however, are inconclusive and currently there is not enough evidence to directly link this virus to incidence of mental disorders.

The distress related to disaster and psychological impacts of quarantine, on the other hand, can be a trigger to ignite preexisting psychological conditions. Social distancing and quarantine can have both short- and long-term effects. Frustration, rage, fear, and anxiety can emerge first. In long term, however, isolation, increased negative symptoms such as social withdrawal, and even developing other comorbid disorders such as posttraumatic stress disorder (PTSD) is possible (Brooks et al., 2020). Inability to obtain medications might be another factor that increase the risk of relapse.

One of the main obstacles of treatment is disruption of psychiatrist–patient relationship. Close human-to-human contact has been declared the main source of transmission of the COVID-19 (Zu et al., 2020). This could vastly affect psychiatrist–patient rapport which is the foundation of a functional treatment (Priebe & Mccabe, 2008). Patients might feel rejected or left alone that cause reduced sense of self-efficacy and helplessness. While utilizing internet have been useful for many people to stay connected with their health care providers and even families and friends, patients with severe mental health problems might face difficulties using technology (Athanasopoulou et al., 2017; Gay et al., 2016; Maguire et al., 2011). Currently, many psychiatrists and psychotherapists visit a number of patients online. This opportunity, however, is not easy to access for many patients with severe mental disorders (Athanasopoulou et al., 2017; Maguire et al., 2011). While someone in therapy can easily change the treatment sessions into the internet based, patients who suffer from cognitive deficits need more practice or sometimes unable to do so. This can increase tension and the burden to the families.

Along with stressors such as fear of infection other factors such as insufficient information and stigma are important concerns. The ability to discriminate fake versus correct news might be diminished in patients who might have preexisting cognitive deficits such as patients with schizophrenia (Lesh et al., 2011). Getting bombarded with instant, indiscriminated and biased messages through social media and internet not only might increase the risk of paranoia in patients, but also might cause hopelessness, panic, and nonproductive behaviors (Maguire et al., 2011; Schrank et al., 2010).

Considerations in patients with severe mental health issues that can increase the risk of relapse are mentioned here:

1. Losing visit appointments and not being able to reschedule another one. Isolation, lack of motivation, and cognitive deficits can lead to less accessibility to mental health providers, especially in areas where community-based practice is not yet well-developed.
2. Incapability of obtaining their medications due to transportation limitations and bans in many regions.
3. Emotional distress and lack of accurate information might cause amplified tension in families. Therefore, the increased “Expressed Emotion” in families might further increase the risk of relapse (Weintraub et al., 2017).
4. Increased negative symptoms such as social withdrawal during the time of quarantine or social distancing.
5. Increased economic pressure due to unemployment, loosing job, or lack of supplies.

There are some suggestions that might be helpful in dealing with the mentioned crisis.

1. Facilitating community-based treatment strategies for patients and families as widely as possible. Arranging telephone follow-ups if other technology-based options seem so complicated or unreachable for the patients (Ho et al., 2020).
2. Supporting and facilitate learning coping strategies while enhancing the feeling of being cared and safe (Alvarez & Hunt, 2005; Brooks et al., 2020). Empathy is the key. Patients and families’ concerns should be heard and validated (Elliott et al., 2011; Fox, 2000).
3. Providing necessary guidelines for patients and their families on how to use social media and internet, for example, where they can find some legitimate information.
4. Simple but useful instructions such as maintain sleeping hygiene to have a good quality sleep, eating well, humor, music, and regular exercise (Marziali et al., 2008; Umlauf & Shattell, 2005).
5. Encourage care-takers and families to include patients in everyday activities while maintaining the necessary social distancing.
6. Empowering families by giving them the sense of self-efficacy and necessary information or training on preventive measures (Marziali et al., 2008).
7. Ensuring that patients have adequate provisions and medications.
8. If possible, assigning someone with adequate information as the “trustee” for the patient, so in case of any question or problem, the patient can refer to him or her.

This is not the first pandemic nor would it be the last one. Maybe it is time for us to focus on integration of e-Treatment in psychiatry, develop e-treatment facilities, and face the challenges more rigorously. Advances in technology are enormous and psychiatric patients should benefit from these advances as much as others and it is our responsibility to keep our treatment strategies up-to-date and not only treat patients, but foresee what future might hold, be prepared and implement predictive measures. Adopting to the new remote treatment strategies might take time and energy, however, it seems necessary more than ever.
